# The cancer stem cell model: B cell acute lymphoblastic leukaemia breaks the mould

**DOI:** 10.1002/emmm.201202207

**Published:** 2012-12-11

**Authors:** James Scott McClellan, Ravindra Majeti

**Affiliations:** Department of Medicine, Division of Hematology, Institute for Stem Cell Biology and Regenerative Medicine, and Cancer Institute, Stanford University School of MedicinePalo Alto, CA, USA

**Keywords:** ALL, cancer stem cells, leukaemia, leukaemia propagating cells, leukaemia stem cells

See related article in EMBO Molecular Medicine http://dx.doi.org/10.1002/emmm.201201703

The cancer stem cell (CSC) hypothesis postulates that tumours are arranged in a developmental hierarchy reflecting heterogeneity on the cellular level. Within a given tumour, only a subset of cells have the capacity to self-renew, give rise to more differentiated progeny and maintain the tumour long-term (Nguyen et al, 2012). The model implies that cancer therapy must eliminate all CSCs in order to achieve a cure.

The existence of CSCs was first demonstrated in human acute myeloid leukaemia (AML) using xenotransplantation experiments in immunodeficient mice. Subsequent work has demonstrated that CSCs in most cases of AML comprise a subpopulation of cells that express several cell surface markers in common with normal hematopoietic stem and progenitor cells, as well as other leukaemia stem cell (LSC)-specific surface markers (Majeti, 2011). AML LSC appear to enter the cell cycle infrequently, making them relatively resistant to standard chemotherapeutic agents that are cell cycle-dependent. Currently, considerable effort is being directed at the development of novel therapies to clear LSC from AML patients, with the hope that this will prevent relapse and lead to cures.

Like AML, precursor B-acute lymphoblastic leukaemia (B-ALL) is thought to develop from malignant transformation of immature hematopoietic progenitor cells. By analogy to AML, it has been postulated that a rare LSC population must exist in B-ALL. However, despite considerable investigative effort, prospective isolation and characterization of B-ALL LSC has remained elusive (Bernt & Armstrong, 2009). Several groups have reported the isolation of B-ALL LSC, but the phenotype of these cells has been inconsistent across multiple studies. Indeed, some studies have indicated that B-ALL blasts retain leukemogenic potential across multiple stages of immunophenotypic maturation.

In this issue of EMBO Molecular Medicine, Rehe et al (2013) build on earlier work in which they demonstrated that in high-risk B-ALL, leukemogenic potential was retained in CD34^+^/CD19^−^ (the least mature subset as judged by immunophenotype), CD34^+^/CD19^+^ and CD34^−^/CD19^+^ populations (le Viseur et al, 2008). In the current study, they again use a xenograft model to identify cell populations capable of establishing leukaemia in immunodeficient mice. They extend their previous analysis by including both standard-risk and high-risk B-ALL cases with a variety of chromosomal abnormalities, and they largely focus on primary samples that have not been previously expanded in immunodeficient mice. They report that LSC are not enriched in blast populations expressing either CD34 or CD10, both markers of immaturity in the hierarchy of normal B cell development. Additionally, they find that even CD20^+^ B-ALL blasts retain leukemogenic potential. This is surprising given that CD20 is a phenotypic marker of maturity normally expressed only late in B cell development. Further, they find that B-ALL blasts do not seem to be arranged in a hierarchy similar to normal B cell precursors. For example, they find that CD34^−^ blasts can give rise to both CD34^−^ and CD34^+^ populations in NOD/SCID/gamma-null (NSG) mice, and vice versa. This is true of the markers CD10 and CD20 as well.

»…the findings of Rehe et al suggest that B-ALL cells are not arranged in a strict hierarchy…«

Impressively, the authors further utilize limiting dilution xenotransplantation assays to calculate the LSC frequency in the B-ALL samples, and subpopulations isolated based on differential expression of CD10, CD20 or CD34. They find that the LSC frequency does not significantly differ within these defined subpopulations, and moreover, that as many as one in 40 primary B-ALL blasts retain leukemogenic potential, a frequency much higher than reported for AML (Eppert et al, 2011). When one considers that these cells are manipulated *ex vivo* and then must overcome a xenotransplantation barrier, it is likely that the true LSC frequency is higher. Indeed, in some B-ALL cases, most or even all of the leukemic blasts may be functional LSC. Taken together, the findings of Rehe et al (2013) suggest that B-ALL cells are not arranged in a strict hierarchy, and that LSC activity is frequent and cannot be isolated by immunophenotypic markers of normal B cell differentiation.

In the case of AML, LSC were initially isolated based on cell surface marker expression. More recently, AML LSC were shown to have a gene expression profile distinct from the bulk of the leukemic blasts (Eppert et al, 2011; Gentles et al, 2010). Not surprisingly, genes associated with self-renewal were preferentially expressed in AML LSC. With this in mind, Rehe et al (2013) examined the transcriptional profile of CD34^+^ B-ALL blasts and phenotypically more mature CD34^−^ B-ALL blasts. Consistent with their xenograft data, they found no distinction between these two populations with regard to their expression of candidate stem cell genes. Additionally, they show that the CD34^+^ and CD34^−^ populations express similar amounts of the telomerase reverse transcriptase (*TERT*), a component of the telomerase holoenzyme that is required to maintain telomeres during cell division. *TERT* is expressed in normal hematopoietic stem cells; thus, these findings are consistent with the notion that both CD34^+^ and CD34^−^ B-ALL blasts retain stem cell function.

The findings of Rehe et al (2013) contrast with multiple older studies which have found that leukemogenic potential is limited to a subset of B-ALL blasts (Bernt & Armstrong, 2009). These disparate findings may be explained by differences in the xenotransplantation model used. The older studies largely used NOD/SCID mice, while Rehe et al (2013) employ an NSG mouse model. NSG mice are more permissive for engraftment of human hematopoietic cells than NOD/SCID, and this difference may have unmasked the leukemogenic potential of the more mature subsets of B-ALL blasts. Additionally, unlike previous studies, Rehe et al (2013) transferred cell suspensions directly into the bone marrow cavity using intrafemoral injection, a technique that likely allowed these cells better access to a favourable niche.

**Figure 1:**
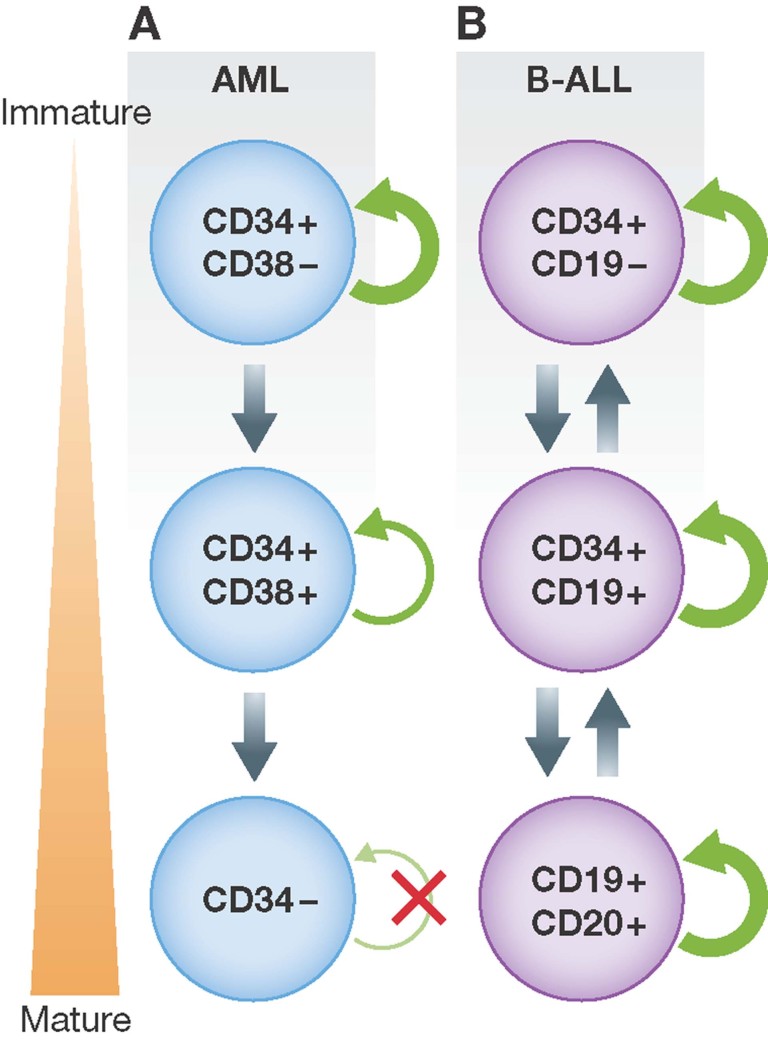
Contrasting cancer stem cell models AML follows a hierarchical model in which more mature blasts lose stem cell capacity.B-ALL may follow a stochastic model by which most, if not all, cells within the tumor are able to transplant the disease in xenograft models, and there is no strict hierarchy.

Taken together, the authors' findings suggest that the hierarchical LSC model of AML does not apply to B-ALL (Fig 1). The authors propose the various immunophenotypically defined subpopulations seen in B-ALL do not differ in a biologically meaningful way, but rather represent stochastic differences in gene expression. They further propose that most, if not all, B-ALL blasts are capable of recapitulating a tumour under the right circumstances. The clinical relevance of these findings is unclear, but they do suggest that therapies designed to target only a subset of B-ALL blasts will not be successful at curing patients.

These findings provide significant insights into the pathogenesis of B-ALL, yet also raise many new questions. For example, if B-ALL subpopulations are not distinct with regard to their leukemogenicity, why do they differ immunophenotypically? Do immunophenotypically defined subpopulations differ in other properties, for example chemoresistance? What do these findings tell us, if anything, about the cell of origin in B-ALL? Does the plasticity in immunophenotype reflect an underlying plasticity of B-ALL blasts? Most fundamentally, why does AML maintain a hierarchy similar to normal hematopoiesis while B-ALL does not appear to follow such a paradigm?
